# Comparison of the Antibacterial Efficacy of* Commiphora molmol* and Sodium Hypochlorite as Root Canal Irrigants against* Enterococcus faecalis* and* Fusobacterium nucleatum*

**DOI:** 10.1155/2019/6916795

**Published:** 2019-07-04

**Authors:** Ebtissam M. Al-Madi, Amal A. Almohaimede, Mohammad I. Al-Obaida, Amani S. Awaad

**Affiliations:** ^1^Department of Restorative Dental Sciences, College of Dentistry, King Saud University, P.O. Box 60169, Riyadh 11545, Saudi Arabia; ^2^Gateway to UK Education, Bradford, UK

## Abstract

**Objective:**

The investigation aims to compare antimicrobial efficacy of the extract of* Commiphora molmol*, against* Enterococcus faecalis* and* Fusobacterium nucleatum*, with sodium hypochlorite (NaOCl).

**Design:**

T he dehydrated oleo-gum resin of* Commiphora molmol* was extracted by using 70% ethanol and was suspended in 99.8% dimethyl sulfoxide (DMSO) as a dissolving agent in a 1:2 volume to produce an aqueous solution at room temperature. Agar-well diffusion and broth microdilution methods assay were utilized to determine both the antimicrobial activity and minimum inhibitory concentration, of alcoholic extract of* Commiphora molmol* against* E. faecalis* and* F. nucleatum*. The values of the inhibition zones were determined based on the concentration of the investigated material. One hundred and forty extracted human premolar teeth were instrumented and immersed in bacterial suspension of* E. faecalis* or* F. nucleatum* (70 teeth in each species suspension). Prepared teeth were then immersed in the myrrh extract solution, 2.5% NaOCl, DMSO, or Cefotaxime and incubated for 30 and 60 minutes.

**Results:**

The largest inhibition zone diameter for both bacterial species was obtained by the 100mg/100*μ*L concentration. The minimum inhibitory concentration (MIC) was 0.03mg/300*μ*L for both* E. faecalis* and* F. nucleatum*. The minimum bactericidal concentration (MBC) results showed that 0.03mg/*μ*L myrrh extract and 2.5% sodium hypochlorite significantly reduced bacterial growth at both 30 and 60 minutes of different treatments of root canals, compared to DMSO group (negative control) and the antibiotic group (positive group).

**Conclusion:**

Myrrh extract was proven to have considerable antibacterial activity against both* F. nucleatum *and* E. faecalis*.

## 1. Introduction

Endodontic infections are characterized by being polymicrobial with the domination of obligate anaerobic bacteria [1].* Enterococcus faecalis* is a facultative anaerobe which is predominant in persistent infections after root canal treatment with a prevalence of 29%-77% [2].* Fusobacterium nucleatum* is one of the most prominent bacteria found in dentinal tubules of roots of nonroot canal treated teeth with apical lesions [3].

Sodium hypochlorite (NaOCl) is the golden standard for irrigants for chemomechanical debridement of root canals due to its antimicrobial action in addition to its exceptional capacity to dissolve remnants of necrotic tissue [4]. Although the use of 5.25% NaOCl for biomechanical preparation of root canals has been determined as an optimum antibacterial agent, it has been considered cytotoxic if injected beyond the apical foramen [5]. It also has an unpleasant smell and taste, a possibility of causing corrosion [6], and may trigger allergic reactions [7]. In addition, it could harm permanent tooth follicles, oral mucosa and peripheral tissues [8]. At lower concentrations, NaOCl provoked fibroblast cytotoxicity in concentrations greater than 0.05% [9]. Therefore, an effective, yet safer, alternative for NaOCl would be preferred.

Myrrh is an aromatic oleo-gum resin acquired as an exudate from the trunk of* Commiphora myrrha* or* Commiphora molmol* (family Burseraceae) [10], which are small trees with short sharp branches that grow in sand and rocky areas in Ethiopia, Somalia, Sudan, Saudi Arabia, Kenya, Yemen, North East Africa, and the Middle East. Myrrh consists of 57-61% water-soluble gum, 25-40% alcohol-soluble resin, 7-17% volatile oil, and 3-4% impurities [11]. The gum comprises proteins and polysaccharides whereas the volatile oil is constituted of sterols, steroids, and terpenes [12]. Historically, myrrh had been used to treat inflammations and infections [10]. In modern times, myrrh has shown an anti-inflammatory effect against both chronic and acute inflammation [13]. It also has antimicrobial activity against staphylococci [10, 14],* Pseudomonas aeruginosa, Escherichia coli*, and* Candida albicans *[10]. It has proven that it is safe for humans even after long-term use [15]. There are no studies that show that myrrh has an antibacterial effect against organisms that have a role in the etiology of persistent periradicular lesions subsequent to root canal treatment.

The aim of this experiment was to assess the antimicrobial activity of myrrh against* E. faecalis* and* F. nucleatum, in vitro*, as compared to sodium hypochlorite in extracted teeth.

## 2. Materials and Methods

### 2.1. Preparation of Myrrh Extract and Bacterial Species

Fifty grams of myrrh resin was extracted by percolation in 500 mL of ethanol (95%). The entire ethanol extract was concentrated using reduced pressure at a temperature that did not exceed 35°C to produce a dry extract of 20g of total extracts [16] and was suspended in 99.8% dimethyl sulfoxide (DMSO) (Atlantic Research Chemicals Ltd., Cornwall, UK) as a dissolving agent in a 1:2 volume to produce an aqueous solution at room temperature.

*E. faecalis* strain (ATCC 29212) and* F. nucleatum*(ATCC 25586) (Watin-Biolife, Riyadh, KSA) were cultured on brain heart infusion (BHI) broth (Thermo Scientific Oxoid Microbiology Products, Hampshire, UK) and agar plates (IBNSINA Medical Factory, Sitrah, Bahrain) in anaerobic jars and incubated for 24 hours at 37°C. Anaerobic conditions were generated through the use of a gas generating kit (GaPak, Oxoid Ltd., Basingstoke, Hampshire, UK). A single colony was inoculated into 5 mL of BHI broth and grown overnight at 37°C [17].

### 2.2. Antibacterial Susceptibility Test

An agar-well diffusion method was used to investigate the antimicrobial activity of myrrh extract and 2.5% NaOCl. BHI agar plates were prepared and bacterial cultures were grown on them. Five wells (4 mm in diameter and 5 mm in depth) were made in each of the agar plates. Two hundred microliters of different concentrations of myrrh extract (5mg extract/100*μ*L DMSO, 10mg extract/100*μ*L DMSO, 50mg extract/100*μ*L DMSO, and 100mg extract/100*μ*L DMSO) were used to fill 4 wells, and the fifth well was filled with 200*μ*L of Dimethyl sulfoxide (99.8% DMSO) as a control. The plates were incubated for 24 hours at 37°C. The plates were removed after the incubation period and measurement of the zones of inhibition were taken. The diameter (mm) of microbial inhibition zones surrounding the wells that contain the test material was measured using a caliper and documented. The inhibitory zone was measured as the shortest distance from the initial point of microbial growth to the outer margin of the well.

### 2.3. Minimum Inhibitory Concentration (MIC)

The MIC of myrrh extracts against* E. faecalis* and* F. nucleatum* were determined by microdilution assay [17]. Fifty microliters of 3×10^8^ of bacteria (1 McFarland turbidity), 50*μ*L of BHI broth, and 100*μ*L of serial dilutions of myrrh extract were added to each well of a 96-well microplate, for a total of 200*μ*L/well. A tenfold series of concentrations of 300mg/100*μ*L, 30mg/100*μ*L, 3mg/100*μ*L, 0.3mg/100*μ*L, and 0.03mg/100*μ*L of extract solutions were made in BHI [18]. Twelve wells were used for each dilution. For comparison to NaOCl, twelve wells were used to inoculate 100*μ*L of 2.5% NaOCl with 50*μ*L bacterial suspension and 50*μ*L BHI for each well. To evaluate the effect of the suspension, 100*μ*L of 99.89% DMSO and 50*μ*L BHI broth were added to each of twelve wells and inoculated with 50*μ*L bacterial suspension. For the positive control, three wells were used to inoculate each well with 200*μ*L of bacterial suspension. For the negative control, three wells were inoculated with 200*μ*L of BHI in each well. A separate microplate was used for each bacterial species assessed. The microplates were incubated for 24 h at 37°C. The optical density of each well was evaluated after incubation using a spectrophotometer (MTX Lab Systems, Inc, Virginia, USA) at 600 nm before and after plater incubation in anaerobic jars at 37°C for 24 hours. The minimum inhibitory concentrations (MIC) of the extract that repressed the visible growth of* E. faecalis* and* F. nucleatum* were determined.

### 2.4. Minimum Bactericidal Concentration (MBC)

The MBC was decided by subculturing of the wells that displayed no perceivable growth on a sterile agar plate. Twenty-five microliters of the bacterial solutions that was considered as the MIC and higher concentrations was grown on BHI agar plates. Six agar plates were used for each concentration. The plates were incubated in anaerobic jars for 24 hours at 37°C. Anaerobic conditions were generated through the use of a gas generating kit. Each plate was examined for growth at the conclusion of the incubation period, both by the naked eye and by using a digital camera to take photographs (Canon IXUS 9015, Canon Inc., Japan). Colony forming units (CFUs) were calculated on a grid. The MBC value was concluded as the lowest concentration that showed no apparent growth on agar subculture.

### 2.5. Teeth Selection, Preparation, and Contamination

One hundred and forty extracted sound single-rooted human premolar teeth with noncalcified, single canals, roots free from resorption, and caries, with mature apices were selected. Teeth were cleaned and then decoronated to a length of 14 mm length. The working length was established by retracting a size 15 K-file (Maillefer) that was inserted into the canal, once the tip was evident at the apical foramen. K3 nickel-titanium rotary files (SybronEndo, California, USA) rotating at a speed of 300 rpm at a torque-control level of 3 were used to instrument the root canals with crown-down methodology. The samples were irrigated with a total of 10 ml of 5.25% NaOCl (Parcan, Specialties Septodont, Saint-Maur-Des-Fosses, France) for 5 minutes then by 3 ml of 17% EDTA for 1 minute as a concluding rinse to remove inorganic and organic debris. To inactivate the effect of NaOCl, 10% sodium thiosulfate (Scharlau, Barcelona, Scharlab S.L, Spain) was injected into the roots. The apical foramen was sealed with GIC (Fuji II LC, GC Corporation, Tokyo, Japan) to prevent bacterial leakage, and the outside surfaces of the root samples were sealed with two layers of cyanoacrylate material (SuperGlue, Alteco Chemical Pte Ltd., Indonesia). The samples were thoroughly rinsed with H_2_O and autoclaved (2100 Classic Autoclave Distillation Units, Absolute Medical Equipment, NY, USA) for 20 minutes at 126°C and 15 lb of pressure. To confirm sterility, teeth were cultured in BHI broth for 24 hours at 37°C [19]. Ten ml of* E. faecalis *and* F. nucleatum *suspensions containing 3 X 10^8^ CFU mL^−1^ was used to contaminate the randomly divided tooth samples and then incubated for 72 hours at 37°C (n=70 for each bacterial species).

Following incubation, the first microbial sampling (S1) was done by flooding the canal with sterile 0.05 mol/L phosphate-buffered saline (PBS) (Pharmaceutical Solutions Industry, Jeddah, Saudi Arabia) at a pH of 6.8 and then inserting a size 50 H-file (Dentsply Maillefer, Tulsa, OK) within the canal shorter 1 mm of the working length to scrape the dentin at the same time. Three sterile absorbent paper points (Meta Biomed Co., Ltd., Korea) were inserted in the canal for 60 seconds and then transferred into test tubes that contain 1.0 ml PBS. The contents of each canal were serially diluted and plated on BHI agar plates. Colony count on each plate was performed after being incubated at 37°C anaerobically for 24 hours [19]. Both bacterial groups were randomly assigned to the following four subgroups*: *myrrh ethanol extract (30mg/300uL, n=30), 2.5% NaOCl (n=30), DMSO (n= 5) as negative control, and 1g of Cefotaxime antibiotic injection as positive control (n= 5). Materials were injected into the prepared root canals in a volume of 10 ml for each tooth by using a 30-gauge side-vented needle (Monoject, Covidien LP, Deland, FL) inserted to 1mm above the apical seal. Agitation was performed with sterile gutta-percha cones (SureDent Corporation, Gyeonggi-do, Korea) for three minutes. Teeth were incubated for 30 minutes or 60 minutes at 37°C. After incubation, the second microbial sampling (S2) and the colony count were performed again for each incubation time, by examining each plate for growth by the naked eye, and photographed by a digital camera (Canon IXUS 9015, Canon Inc. Japan). The images were examined and viewed in the computer monitor. CFUs were calculated from the image magnified on the monitor.

The percent in colony count reduction prior and after application of test agents was calculated utilizing the formula [19]

(1) “Percentage  reduction  in  colony”=“Initial  colony−Final  colony  count”Initial  colony  count  X  100

### 2.6. Statistical Analysis

The data was collected, and the four concentrations were compared to each other at two-time points by One-way Repeated Measures ANOVA test followed by Tukey's multiple comparison tests with a significance level of 5%. Paired t-test was used to compare the two-time points in each concentration. Version 16 of SPSS program was used.

## 3. Results

The findings of this study showed that myrrh extract has antibacterial activity against* E. faecalis* and* F. nucleatum*. In* E. faecalis* group, the highest inhibition zone diameter was obtained by the 100mg/100*μ*L concentration (20mm), while the DMSO group showed an inhibition zone diameter of 1.5 mm. The* F. nucleatum* group showed that the highest inhibition zone diameter was also obtained by the 100mg/100*μ*L concentration (12mm), compared to the DMSO group, which showed an inhibition zone diameter of 9.5mm. In the* F. nucleatum *bacterial group, a highly statistically significant difference in the mean absorbance scores across the different dilutions used at both points of observation (preincubation and 24-hour postincubation) (Table 1) was found. In the* E. faecalis *bacterial group, at the preincubation period, the mean absorbance of media, DMSO, 2.5% NaOCl, and 300mg/300uL of myrrh extract was much lower than the mean absorbance scores of other dilutions (30, 3, 0.3, and 0.03 mg/300uL), although this difference has not reached statistical significance (Table 2). Both MIC and MBC were determined as 30mg/300*μ*L for both* E. faecalis* and* F. nucleatum*.

The percent reduction in colony counts showed that 30mg/*μ*L myrrh extract and 2.5% sodium hypochlorite significantly reduced both bacterial growth at 30 and 60 minutes of treatment in root canals, with statistical significance (p<0.05), compared to the negative control (DMSO group) and the positive group (antibiotic group).

## 4. Discussion

The utilization of herbal therapies and natural products has increased significantly in many disciplines including dentistry. Numerous plants were investigated with regard to their potential as an antimicrobial agent in endodontic infections. Several of these plants/plant products are propolis,* Morinda citrifolia, Arctium lappa, Liquorice, Triphala, Syzygium aromaticum, Ocimum sanctum Green Tea Polyphenols,* and* Cinnamomum zeylanicum* [20–25]. This study investigated the potency of* Commiphora molmol *(myrrh) to be used as an irrigant in endodontic therapy.

The results showed that* Commiphora molmol* (myrrh) extract has antibacterial activity against* E. faecalis* and* F. nucleatum*. Moreover, the myrrh extract significantly reduced the colony forming units of* E. faecalis* and* F. nucleatum.*

The promising antimicrobial activity of myrrh shown in the MIC assay was in agreement with previous studies reporting antistaphylococcal activity of* Commiphora molmol* [10, 14] and antimicrobial activity against* Pseudomonas aeruginosa*,* Escherichia coli,* and* Candida albicans* [10]. Other natural products also showed antimicrobial activity as* Commiphora molmol* against several bacterial species; liquorice extract had a potent antibacterial effect against* E. faecalis* and* Streptococcus mutans *[24].* Salvadora persica *(Miswak extract) also showed antibacterial effect on* Porphyromonas gingivalis*,* Aggregatibacter actinomycetemcomitans*,* Haemophilus influenzae*,* Streptococcus mutans,* and* Lactobacillus acidophilus *[26]. Moreover, Morinda Triphala, Citrifolia juice, and green tea polyphenols displayed an antibacterial effect against* E. faecalis *[22, 23]. In addition, propolis demonstrated antimicrobial action against* Haemophilus influenzae, Streptococcus pneumoniae*, Moraxella* catarrhalis*, cocci, and Gram-positive rods [27].

In the current study, promising antimicrobial activity of myrrh was shown in both MIC assay and MBC assay against both bacteria used. These tests are the parameters generally used for evaluation of the activity of an antibacterial agent. Several previous studies showed strong antimicrobial activity of* Commiphora molmol* [10, 14, 28].

In this study, teeth were autoclaved before use. Several studies showed that autoclaving showed no effect on dentin permeability and bond strength [29]. Moreover, it is simple, inexpensive, and appropriate for routine use in research purposes [30]. Time periods that were used in this section of the study were *½*  hour and 1 hour to mimic the clinical situation where the disinfecting solution during endodontic treatment visit stays in contact with pulp tissue for *½*  hour or 1 hour; therefore, the effect of myrrh extract was assessed during those durations. The current results presented showed that there was no significant difference among the mean percentage reduction for both bacteria groups (*F. nucleatum *and* E. faecalis) *in the myrrh group, NaOCl group, and the antibiotic group (positive control) in both time periods (*½*  hour and 1-hour posttreatment). However, each type of bacteria responded differently to the myrrh extract, which could be attributed to the species differences of these bacteria.* E. faecalis* is a Gram-positive bacterium with a dense peptidoglycan film that acts as a barrier to many synthetic and natural antibiotics, while* F. nucleatum* bacteria are a gram-negative bacterium with thin peptidoglycan layer [31], besides the innate and acquired adaptive properties that characterize* E. faecalis* bacteria [32].

Phytochemical screening of myrrh has identified the existence of primary and secondary metabolites [33, 34]. The antimicrobial effect of myrrh can be attributed to the secondary plant metabolites [34]. Myrrh contains phenols that exert strong antimicrobial action against several bacteria including* E. faecalis* [35]. The antimicrobial action of phenolic compounds are characterized by binding to nucleophilic amino acids in bacterial proteins, which leads to inactivation of the protein and loss of its function, therefore prevention of the bacterial life cycle [36]. Furthermore, phenolic compounds target the surface exposed adhesins in the microbial cell, which will prevent bacterial adherence with other cells and formation of biofilms [37]. Alkaloids, present in myrrh, have also shown antimicrobial activity against* E. faecalis* [38]. Alkaloids exhibit their antimicrobial action by inhibition of protein biosynthesis in bacteria as in phenolic compounds and by changing the permeability of bacterial biomembranes [39]. Shen (2012) showed that sesquiterpenoids were responsible for the antimicrobial effects of myrrh. This author also reported that the resin of* Commiphora molmol* was beneficial for treatment of diseases caused by microbial infections. The toxicity of Commiphora species has been shown to be limited, only induced by large quantities and mainly involving its volatile oil, causing allergy, nausea, and decreased locomotor ability [40].

Within the limitation of the present study, different concentrations of myrrh showed promising results of antimicrobial activity against* F. nucleatum* and* E. faecalis* bacteria that are specific for the oral cavity and root canals, especially after 24 hours with dilutions of 30mg and 0.03mg. We also conclude that there was no significant difference in the antimicrobial effect of 30mg/300*μ*L myrrh from that of sodium hypochlorite (NaOCl) against both bacterial species. Myrrh may be an effective antimicrobial agent that may be used as an intracanal medicament, irrigant, or final rinsing agent. However, further studies are recommended, before its use, regarding its effectiveness in root canals against biofilms, its biocompatibility, and its ability to eradicate the dentinal smear layer.

## Figures and Tables

**Table 1:** One-way ANOVA for the optimum concentration of myrrh extract that gave maximum antimicrobial effect against* Fusobacterium nucleatum *bacteria at 5% significant level.

*Dilutions of myrrh extract and controls*	*The mean absorbance and standard deviation (SD) at different observation periods*	
	Pre-incubation	24 hours post -incubation
300mg/300uL (1:1)	0.54± 0.31^*∗*^	0.49± 0.31
30mg/300uL (1:10)	1.04± 0.43^*∗*^	0.08± 0.07^*∗*^
3mg/300uL (1:100)	1.51± 0.13	0.60± 0.21
0.3mg/300uL (1:1000)	1.56± 0.06	0.16± 0.21^*∗*^
0.03mg/300uL (1:10000)	1.50± 0.03	0.19± 0.24^*∗*^
DMSO	0.08± 0.02	0.09± 0.02
Bacteria	0.24± 0.01	0.88± 0.08
Media (-ve control)	0.06± 0.01	0.20± 0.18
2.5% NaOCl	0.12± 0.06	0.14± 0.05

*F-value*	100.53	16.60

*p-value*	<0.0001*∗*	<0.0001*∗*

*∗*Significant at P value < 0.05.

**Table 2:** One-way ANOVA for the optimum concentration of myrrh extract that gave maximum antimicrobial effect against* Enterococcus faecalis *bacteria at 5% significant level.

*Dilutions of myrrh extract and controls*	*The mean absorbance and standard deviation (SD) at different observation periods*	
	Pre-incubation	24 hours post -incubation
300mg/300uL (1:1)	0.33± 0.45^*∗*^	0.23± 0.25^*∗*^
30mg/300uL (1:10)	1.21± 0.28	0.20± 0.09^*∗*^
3mg/300uL (1:100)	1.49± 0.14	0.34± 0.09
0.3mg/300uL (1:1000)	1.54± 0.08	0.41± 0.09
0.03mg/300uL (1:10000)	1.52± 0.03	0.23± 0.11^*∗*^
DMSO	0.09± 0.01	0.09± 0.02
Bacteria	0.25± 0.02	0.83± 0.07
Media (-ve control)	0.06± 0.002	0.32± 0.33
2.5 % NaOCl	0.11± 0.03	0.13± 0.02

*F-value*	109.64	15.63

*p-value*	<0.0001*∗*	<0.0001*∗*

*∗*Significant at P value < 0.05.

## Data Availability

Data can be provided upon request.
